# Recruitment maneuvers for acute respiratory distress syndrome: the panorama in 2016

**DOI:** 10.5935/0103-507X.20160023

**Published:** 2016

**Authors:** Pedro Leme Silva, Paolo Pelosi, Patricia Rieken Macêdo Rocco

**Affiliations:** 1 Laboratory of Pulmonary Investigation, Instituto de Biofísica Carlos Chagas Filho, Universidade Federal do Rio de Janeiro - Rio de Janeiro (RJ), Brazil.; 2 Department of Surgical Sciences and Integrated Diagnostics, Anesthesia and Intensive Care, IRCCS AOU San Martino-IST, University of Genoa - Genoa - Italy.

A recruitment maneuver (RM) uses a dynamic and transient increase in the transpulmonary pressure (the difference between the airway and pleural pressures) to open non-aerated or poorly aerated lung areas.^(1)^ RMs can improve lung mechanics and oxygenation^(2,3)^ but may also temporarily exacerbate epithelial and endothelial cell damage, increasing alveolar-capillary permeability.^(4)^ The use of RMs in patients with acute respiratory distress syndrome (ARDS) is still being debated.^(1)^ In experimental ARDS, "slow" RM has been found to yield a more homogeneous inflation of the lungs and to reduce functional impairment of the lungs to a greater degree than "fast" RM (a continuous positive airway pressure of 30cmH_2_O for 30 seconds) regardless of the etiology^(3)^ and severity of ARDS.^(5)^ It was also associated with fewer ventilator-induced lung injuries. Recently, a prospective, multicenter, pilot-scale, randomized controlled trial compared the ARDS Network protocol using low levels of positive end-expiratory pressure (PEEP) with an open lung approach (RM and decremental PEEP trial), which resulted in moderate to high PEEP levels for the management of moderate/severe ARDS. The authors observed improvements in oxygenation and driving pressures with the open-lung approach but found no effects on 60-day mortality or ventilator-free days. Following these results, the authors suggested the initiation of a large multicenter trial.^(6)^

To evaluate the beneficial effects of RMs, several parameters should be measured in addition to oxygenation and compliance, such as driving pressure^(7)^ and mechanical power. Driving pressure values higher than 15cmH_2_O result in higher mortality in patients with ARDS. More recently, the concept of mechanical power has been introduced as a parameter to monitor ventilator-induced lung injury development in healthy lungs, with one report indicating that mechanical power levels higher than 12J/min are associated with lung injury.^(8)^

Lung RMs have been used not only in the context of ARDS but also in non-injured patients undergoing surgery in order to reverse anesthesia-induced atelectasis. Nevertheless, to date, there has been no clear indication that RMs can prevent postoperative pulmonary complications (PPCs). One recent meta-analysis^(9)^ of data from randomized controlled trials pooled 2,250 non-injured patients who received protective ventilation to determine whether tidal volume, PEEP, and driving pressure were associated with PPCs. The authors observed that driving pressure during intraoperative ventilation was independently associated with the development of PPCs after surgery. Therefore, it is not the effect of lung RMs *per se* that may lead to beneficial or harmful effects. Instead, RMs should be considered tools to reduce driving pressure and power and to maintain these parameters within a protective range. Increasing the PEEP level for a short period can lead to divergent changes in driving pressure. If the increase in the PEEP level leads to increased aeration of the lung tissue through recruitment, a decrease in driving pressure is expected. On the other hand, if the PEEP increases and does not recruit lung tissue, the lung may become overstretched, and the driving pressure may remain unchanged or even increase over time. Driving pressure appears to be an important parameter for the optimization of mechanical ventilation in non-injured^(10)^ and injured^(11)^ lungs, as well as for intraoperative ventilation.^(9)^ Interestingly, this concept has also been observed at the cellular level. In cell cultures, peak amplitude deformation (force) was not associated with cell death; however, an increase in amplitude (driving) was associated with the worst-case scenario.^(12)^ Parameters other than driving pressure are available to estimate the lung area amenable to ventilation. Beitler et al. used measurements of the maximum insufflation volume (V_RM_) to evaluate lung recruitment.^(13)^ The authors demonstrated that V_RM_ predicted both tidal and end-inspiratory lung stress; they also showed an association of this parameter with 28-day mortality. However, V_RM_ does not clearly indicate if the alveolar opening distribution is homogeneous. Thus, this parameter could include some degree of hyperinflation, leading to increased driving pressure or lung recruitment and decreased driving pressure. In summary, the correlation between driving pressure, V_RM_, and mortality after RMs in ARDS requires elucidation.

Different mechanical ventilation modes can also contribute to lung recruitment. Variable ventilation has been shown to result in greater lung recruitment and lung epithelial cell protection compared to a protective ventilation strategy.^(14)^ Assisted mechanical ventilation is associated with homogeneous lung recruitment, but, depending on the recruitability of the lung, it may also result in deleterious effects.^(15)^ Additionally, assisted mechanical ventilation may exacerbate lung injury by increasing patient-ventilator asynchrony and rapid, shallow breathing.^(15)^ Airway pressure release ventilation^(16)^ has been shown to be effective for lung recruitment in experimental ARDS^(17)^ and in a meta-analysis of trauma patients.^(18)^

## CONCLUSION

Even though experimental studies, systematic reviews, and meta-analyses have suggested that RMs are associated with beneficial effects for lung function and morphology in ARDS, their impact on clinical outcomes is still being debated. Different methods with different benefit and risk profiles have been used to recruit the lungs, and further studies are required to identify the optimal RM method. Lung mechanical parameters associated with ventilator-induced lung injury, such as driving pressure, energy, and mechanical power, have been evaluated recently and should be used to assess the beneficial effects of lung recruitment and the outcomes of ARDS patients. The etiology, severity, and timing of ARDS need to be considered before choosing to recruit the lungs. In this context, the realization that a specific lung area can be opened may reduce the indiscriminate use of RMs for ARDS, as not all lungs are recruitable and, depending on the RM technique used, further lung damage may occur. Additionally, some ventilatory approaches (e.g., variable ventilation and airway pressure release ventilation) can safely recruit ARDS-affected lungs in ways that may minimize pulmonary damage and improve outcomes.
